# Challenges in diagnosing pediatric malaria in Dar es Salaam, Tanzania

**DOI:** 10.1186/1475-2875-11-S1-P92

**Published:** 2012-10-15

**Authors:** Gro EA Strom, Christel G Haanshuus, Sabrina Moyo, Maulidi Fataki, Nina Langeland, Bjørn Blomberg

**Affiliations:** 1 Institute of Medicine, University of Bergen, Bergen, Norway; 2 Muhimbili University of Health and Allied Sciences, Dar es Salaam, Tanzania; 3 Department of Medicine, Haukeland University Hospital, Bergen, Norway

## Background

Malaria is a major cause of pediatric morbidity and mortality. No clinical features clearly differentiate malaria from other causes of pediatric febrile illness. Lack of laboratory equipment and expertise makes malaria diagnostics a challenge, leading to overdiagnosis and overtreatment of malaria. Molecular methods (PCR) are emerging but have so far not proven practical for routine clinical use. Emerging antimalarial and antibiotic resistance calls for precise diagnostics and treatment of febrile illness, and has led WHO to recommend laboratory confirmation of malaria in children before starting treatment.

## Methods

Children admitted with fever were recruited consecutively among admissions at the general pediatric wards at Muhimbili National Hospital (MNH) in Dar es Salaam Tanzania from January-June 2009. Clinical, demographic and laboratory features were registered. Microscopy of thick blood smears was done as part of the routine at MNH, and thin blood smears were stained and examined later. Retrospectively, genus-specific PCR of Plasmodium mitochondrial DNA was performed on DNA extracted from whole blood for all patients and species-specific PCR was done on samples positive by genus-specific PCR. Univariate and multivariate statistical analysis was performed using IBM SPSS Statistics version 19 (SPSS Inc, IBM Company).

## Results

The study included 304 children. Within four weeks before admission 62.6% had received antimalarials. Forty children had positive routine thick blood smears upon admission and twenty had positive research thin blood smears upon retrospective examination. Twenty-five percent had positive PCR, all positive for *P. falciparum.* PCR results confirmed positive routine microscopy in only 52,5% and research microscopy in 100%. Almost every fifth febrile child (55/304) had positive PCR but negative research microscopy. High parasitemia on routine microscopy was associated with positive research microscopy and positive PCR. The true prevalence of malaria in the population remains unknown as none of the diagnostic methods can be interpreted as a true gold standard. Palmar pallor, low hemoglobin and low platelet count were significantly associated with both positive PCR and positive research microscopy (p<0.001). In hospital, 65.1% received antimalarial treatment. Clinically determined severity of palmar pallor was clearly associated with hemoglobin level.

## Conclusions

The study identified discrepancies between routine malaria microscopy, research malaria microscopy and PCR. Almost half of routine microscopy positive cases were negative on PCR, indicating prevalent overdiagnosis of malaria. PCR was positive for many research microscopy negative cases. This may in part be due to prevalent treatment with antimalarials before admission. Palmar pallor and low hemoglobin levels were predictors for malaria in this study. The current routine diagnostic method for malaria appears to lead to overdiagnosis of malaria and, consequently, overuse of antimalarials. Conversely, children with false positive malaria diagnosis may die because they do not receive treatment for the true cause of their illness. Malaria is still a prominent health issue in Tanzania, and the uncertainty of both clinical and routine laboratory tests may lead to misuse of antimalarials and antibiotics and consequently contribute to emerging drug resistance. Diagnostic algorithms employing new methods such as rapid diagnostic tests (RDTs) may contribute to improving malaria diagnosis and treatment.

